# Evaluation of conventional and point‐of‐care real‐time RT‐PCR tests for the detection of SARS‐CoV‐2 through a pooled testing strategy

**DOI:** 10.1002/jcla.24491

**Published:** 2022-05-09

**Authors:** Ji‐Rong Yang, Chuan‐Yi Kuo, Hsiang‐Yi Huang, I‐Ling Yu, Chih‐Tsun Hsieh, Bao‐Shan Chen, Ming‐Tsan Liu

**Affiliations:** ^1^ Center for Diagnostics and Vaccine Development Centers for Disease Control Taipei Taiwan

**Keywords:** point‐of‐care, pooled testing, real‐time RT‐PCR, SARS‐CoV‐2

## Abstract

**Background:**

The rapid identification and isolation of individuals infected with SARS‐CoV‐2 are fundamental countermeasures for the efficient control of the COVID‐19 pandemic, which has affected millions of people around the world. Real‐time RT‐PCR is one of the most commonly applied reference methods for virus detection, and the use of pooled testing has been proposed as an effective way to increase the throughput of routine diagnostic tests. However, the clinical applicability of different types of real‐time RT‐PCR tests in a given group size remains inconclusive due to inconsistent regional disease prevalence and test demands.

**Methods:**

In this study, the performance of one dual‐target conventional and two point‐of‐care real‐time RT‐PCR tests in a 5‐specimen pooled testing strategy for the detection of SARS‐COV‐2 was evaluated.

**Results:**

We demonstrated the proof of concept that all of these real‐time RT‐PCR tests could feasibly detect SARS‐CoV‐2 from nasopharyngeal and oropharyngeal specimens that contain viral RNA loads in the range of 3.48 × 10^5^ to 3.42 × 10^2^ copies/ml through pooled testing in a group size of 5 with overall positive percent agreement (pooling vs. individual testing) ranging from 100% to 93.75%. Furthermore, the two POC real‐time RT‐PCR tests exhibited comparable sensitivity to that of the dual‐target conventional one when clinical specimens were tested individually.

**Conclusion:**

Our findings support the feasibility of using real‐time RT‐PCR tests developed as a variety of platforms in routine laboratory detection of suspected COVID‐19 cases through a pooled testing strategy that is beneficial to increasing the daily diagnostic capacity.

## Graphical abstract

The performance of one dual‐target conventional and two point‐of‐care real‐time RT‐PCR tests in a 5‐specimen pooled testing strategy was evaluated and shown to feasibly detect SARS‐CoV‐2 from nasopharyngeal and oropharyngeal specimens. The overall positive percent agreements (pooling vs. individual testing) in high, medium, and low viral loads are 100%, 100%, and 100%–93.75% for three platforms of real‐time RT‐PCR tests. The 5‐specimen pooled testing is an effective way to increase the throughput of routine diagnostic test for SARS‐CoV‐2 detection.

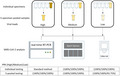

## 1 INTRODUCTION

Coronavirus disease 2019 (COVID‐19) caused by severe acute respiratory syndrome coronavirus 2 (SARS‐CoV‐2) has evolved into a pandemic since its first appearance in December 2019 and has led to more than 386 million confirmed cases and over 5.7 million deaths globally as of February 4, 2022. Currently, nucleic acid amplification tests are the reference standard for the diagnosis of SARS‐CoV‐2 infection, and real‐time RT‐PCR‐based platforms are recommended and have been widely applied.^1^, ^2^ Rapid and accurate laboratory testing for COVID‐19 is important for evaluating the spread of disease, screening unique or at‐risk populations, tracing the contacts of infected individuals, and cutting off epidemic transmission.^3^ Mass testing against SARS‐CoV‐2 and in some circumstances alone with other bacterial, viral, or fungal microbiota is also encouraged for a wide range of COVID‐19 control measures, such as screening for community transmission^4^ and evaluation for disease progression of COVID‐19 patients with/without coinfection of other microbial agents.^5^, ^6^, ^7^, ^8^

Pooling of clinical specimens for mass detection of SARS‐CoV‐2 by nucleic acid testing has been reported to be effective in increasing diagnostic capacity with sufficient accuracy^9^, ^10^, ^11^ for prompt disease intervention, especially when the prevalence of infected cases is low. In Taiwan, routine diagnosis of COVID‐19 among individuals with suspected disease is conducted through a comprehensive nationwide SARS‐CoV‐2 laboratory network that comprises a total of 245 clinical laboratories at regional hospitals and medical institutions with a maximum daily capacity of 149,660 tests as of September 27, 2021.^12^ Real‐time RT‐PCR assays in various types of formats that were given Emergency Use Authorization (EUA) by the Taiwan Food and Drug Administration (FDA) are used, and each specimen needs to be tested individually. To further strengthen the diagnostic capacity for control of the epidemic surge, a pooled testing strategy was adopted in August 2021 for mass detection of SARS‐CoV‐2 in nasopharyngeal and oropharyngeal specimens. The target population applicable to this workflow includes medical personnel, such as healthcare workers, who are compulsorily required to be examined weekly, individuals who are tested at the screening station in the community, and people who are found to have had contact with COVID‐19 cases and need to be examined for SARS‐CoV‐2 infection. The group size of each sample pool is proposed to be 5 for one real‐time RT‐PCR test, taking into account the acceptable detection sensitivity as established by previously published literature.^13^, ^14^ However, there are still concerns regarding the likelihood of false negative results, since the dilution effect derived from pooled testing may impact the tests’ analytical applicability, potentially leading to reduced diagnostic sensitivity.^15^ Furthermore, information on the difference between the performance of point‐of‐care (POC) real‐time RT‐PCR tests, which are widely used by clinical laboratories in Taiwan, to detect SARS‐CoV‐2 from pools of specimens compared to that when the positive specimen is detected individually is scarce.

## 2 MATERIALS AND METHODS

### 2.1 Collection of clinical specimens

The SARS‐CoV‐2 laboratory diagnostic network in Taiwan is well established and is coordinated by the Taiwan Centers for Disease Control (CDC).^12^ Respiratory specimens, including nasopharyngeal and oropharyngeal swabs, were collected from individuals who met the reporting criteria, such as outpatients in the community and hospitalized patients who were suspected to have SARS‐CoV‐2 infection, as well as people who came to Taiwan from abroad. Clinical specimens were then transported to the laboratories of the SARS‐CoV‐2 laboratory network for virus detection by real‐time RT‐PCR.^12^ Original specimens that tested positive in each local laboratory were sent back to the Taiwan CDC for further viral genetic characterization and virus isolation.

### 2.2 Dual‐target conventional and POC real‐time RT‐PCR tests for SARS‐CoV‐2 detection

Conventional real‐time RT‐PCR for SARS‐CoV‐2 detection was defined as the workflow in which viral nucleic acid extraction and detection are performed separately. The dual‐target method that was evaluated in this study was performed as previously reported^16^, ^17^, ^18^ and served as the reference method. Two of the SARS‐CoV‐2 genomic segments, including the envelope (E) and nucleocapsid (N), were the targets of detection in two separate reactions (designate E and N2 assays). Viral RNA was extracted from the clinical specimens using the automated TANBead extraction system (Taiwan Advanced Nanotech) according to the manufacturer's instructions. The Roche LightCycler Multiplex RNA Virus Master Kit (Roche) was used. Each real‐time RT‐PCR assay was performed in a 20‐µl reaction mixture containing 5 µl of RNA template, 8.4 µl water, 0.1 µl RT Enzyme Solution, 4 µl RT‐qPCR Reaction Mix, and a final concentration of 0.5 µM of each primer and 125 nM of probes. The reaction mixtures were incubated at 50°C for 10 min, followed by 95°C for 30 s. The reaction mixtures were then subjected to 45 cycles of 95°C for 5 s, 53°C for 15 s, and 60°C for 15 s. Specimens that tested positive for at least one of the two assays were interpreted as positive.

The POC real‐time RT‐PCR tests evaluated in this study were defined as those that combined viral nucleic acid extraction and detection in a closed system, and the Xpert Xpress SARS‐CoV‐2 (Cepheid) and cobas Liat SARS‐CoV‐2 & Influenza A/B (Roche) tests were selected for evaluation. Each POC test was performed according to the manufacturer's instructions. Xpert Xpress detects the gene sequences of the viral E and N proteins of SARS‐CoV‐2. Cobas Liat detects the viral ORF1a/b and N genes. Interpretation of the test results of each POC real‐time RT‐PCR also followed the manufacturer's instructions.

### 2.3 Generation of the semiquantitative standard curve of the conventional N2 assay for estimation of viral RNA loads in clinical specimens

To determine the viral RNA loads in each SARS‐CoV‐2‐positive clinical specimen taken for evaluation, their cycles of threshold (Ct values) determined by the conventional N2 assay were used for proper estimation. For this purpose, the Amplirun Total SARS‐CoV‐2 Control (Swab) (Vircell) was purchased and served as a reference material to construct the semiquantitative standard curve. Vials of lyophilized inactivated SARS‐CoV‐2 particles were reconstituted in molecular biology grade water, resulting in a final concentration of 35,000 viral RNA copies/ml according to the manufacturer's instructions. Afterward, 2‐fold serially diluted standards with known amounts of viral RNA ranging from approximately 3500–109.375 copies/ml were prepared and subjected to nucleic acid extraction followed by the N2 assay to determine the respective Ct values. A standard curve of the N2 assay was constructed by incorporating the Ct values of each diluted standard tested in 18 replicates.

### 2.4 Preparation of artificial clinical specimen pools

In this study, a 5‐specimen pooled testing strategy for SARS‐CoV‐2 nucleic acid detection by using conventional or POC real‐time RT‐PCR tests was evaluated. The diagnostic performance of one dual‐target conventional and two of the widely used POC real‐time RT‐PCR tests in Taiwan was tested by a panel of artificially spiked nasopharyngeal or oropharyngeal specimen pools containing various SARS‐CoV‐2 RNA loads.

The artificial clinical specimen pools were prepared by spiking one SARS‐CoV‐2‐positive nasopharyngeal or oropharyngeal specimen with four negative ones, each of which was confirmed through routine real‐time RT‐PCR testing at the Taiwan CDC or local laboratories of the diagnostic network. A total of 83 positive specimens were selected according to their original Ct values and retested individually again by the N2 assay. The resultant Ct of each specimen was recorded. We then categorized these positive specimens into three groups representing high, intermediate, and low viral RNA loads based on the N2 Ct values. Furthermore, a total of 76 negative specimens were selected and used for the preparation of pooled clinical specimens in a 1:4 manner (designated 5‐specimen pooled samples).

### 2.5 Statistical analysis

Statistical analyses were performed using a one‐tailed *t* test. Data were considered to be statistically significant at a *p *< 0.05.

## 3 RESULTS

### 3.1 Performance of dual‐target conventional real‐time RT‐PCR for the detection of SARS‐CoV‐2 in the 5‐specimen pooled sample

**FIGURE 1:**
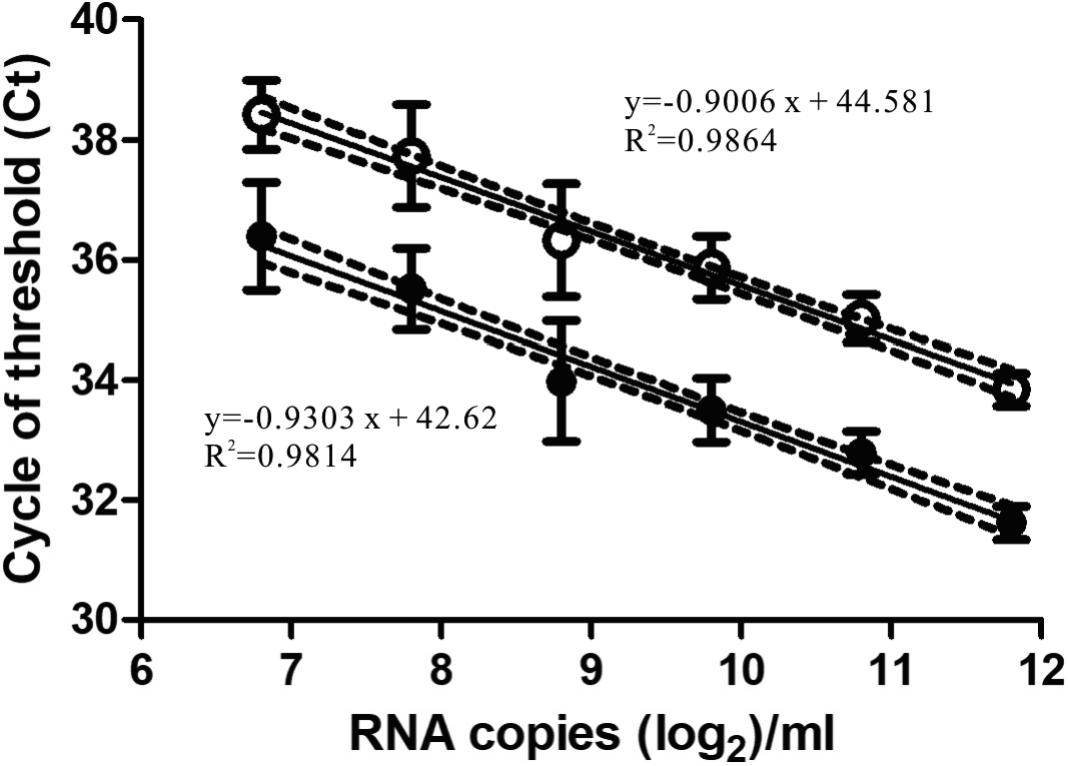
Standard curves for dual‐target conventional real‐time RT‐PCR. Serial twofold dilutions of commercially purchased inactivated SARS‐CoV‐2 particles with known RNA loads ranging from 3500 copies/ml to 109.375 copies/ml were used for RNA extraction followed by real‐time RT‐PCR analysis of (A) E and (B) N2 assays. Each dilution of the standard curve was analyzed by 18 replicates, and the respective mean value is illustrated by dotted and hollow circles, respectively, with standard deviation shown as error bars. The viral RNA loads (log_2_ copies/ml) and the cycles of threshold (Ct) are indicated on the *X*‐ and *Y*‐axes, respectively

To evaluate the performance of the dual‐target conventional real‐time RT‐PCR for the detection of SARS‐CoV‐2 through the pooling strategy, we first determined the semiquantitative standard curve of the E and N2 assays (Figure 1), and the latter was used to estimate the viral RNA loads in a given specimen. A total of the 50 artificially spiked 5‐specimen pooled samples, each of which contained one positive nasopharyngeal or oropharyngeal sample with low (individual N2 assay Ct values of 28–30, *n* = 11), intermediate (N2 Ct of 31–34, *n* = 23), or high (N2 Ct of 35–37, *n* = 16) levels of viruses before pooling, were then prepared for further analysis. The detection results of the 50 positive samples and the corresponding 5‐specimen pooled samples are summarized in Table 1. It showed that dual‐target conventional real‐time RT‐PCR can correctly detect SARS‐CoV‐2 viral RNA through the 5‐specimen pooled testing strategy among all the enrolled positive specimens with viral RNA load levels ranging from 3.48 × 10^5^ to 3.44 × 10^3^ copies/ml before pooling, achieving a positive percent agreement (PPA) of 100% compared to individual testing. For the 5‐specimen pooled samples with low viral loads (1.59 × 10^3^–3.42 × 10^2^ copies/ml) before pooling, 1 out of the 16 had a negative result, and the PPA was 93.75%. The specimen that was missed had Ct values of 34.92 and 37.97 in the E and N2 assays, respectively, when tested individually.

**TABLE 1:** Performance of the dual‐target conventional real‐time RT‐PCR for detection of SARS‐CoV‐2 viruses through the pooled testing strategy

Ct group of individual specimens^a^ (number of specimens)	Viral RNA quantity of the individual specimens (copies/ml)^b^	Results of dual‐target conventional real‐time RT‐PCR through 5‐specimen‐pooled testing		Positive percent agreement (%)^c^
		Positive	Negative	
28–30 (*n* = 11)	3.48 × 10^5^–7.47 × 10^4^	11	0	100
31–34 (*n* = 23)	3.46 × 10^4^–3.44 × 10^3^	23	0	100
35–37 (*n* = 16)	1.59 × 10^3^–3.42 × 10^2^	15	1	93.75

^a^ The Ct group was classified by those determined by the conventional N2 real‐time RT‐PCR assay when the positive specimen in each 5‐pooled specimen was tested individually.

^b^ The amount of viral RNA copies was determined from the Ct value of each individual testing based on the semiquantitative standard curve of the N2 real‐time RT‐PCR assay.

^c^ Positive percent agreement was determined by comparing results of the 5‐pooled specimens with the respective positive specimens detected individually.

**FIGURE 2:**
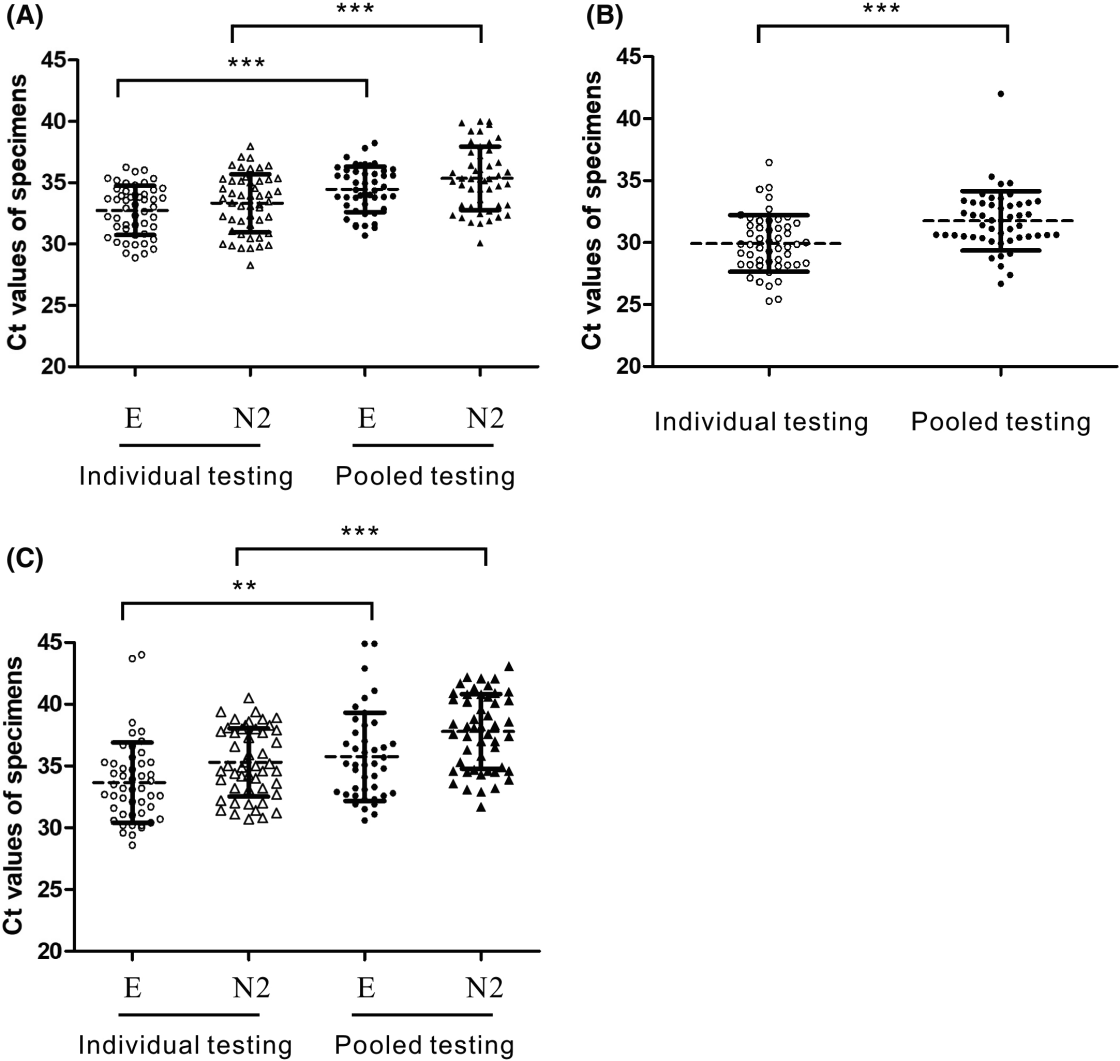
Comparison of Ct values of SARS‐CoV‐2 detection through individual and pooled testing strategies. Data of (A) dual‐target conventional real‐time RT–PCR, (B) Xpert Xpress, and (C) cobas Liat are presented as a scatter plot with mean values ± SD of the same testing strategy. The asterisks (**) and (***) indicate a significant difference (*p* < 0.01 and *p* < 0.0001, respectively) by a one‐tailed unpaired *t* test. The testing strategy and the resultant Ct values are indicated on the *X*‐ and *Y*‐axes, respectively

Comparing the Ct values of the 5‐specimen pooled samples to those of the respective positive specimens that were tested individually showed that the average Ct values obtained through the pooling strategy significantly increased by 1.7 (*p* < 0.0001) and 2.0 (*p* < 0.0001) in the E and N2 assays, respectively (Figure 2A), mirroring the dilution effect caused by the pooled testing.

### 3.2 Performance of POC real‐time RT‐PCR tests for SARS‐CoV‐2 detection from the individual and 5‐specimen pooled samples

To understand the clinical applicability of POC real‐time RT‐PCR tests to detect SARS‐CoV‐2, another two panels of 50 nasopharyngeal/oropharyngeal specimens and the respective 5‐specimen pooled samples were separately examined by the Xpert Xpress and cobas Liat platforms. For individual testing, both the Xpert Xpress and cobas Liat had identical results to those of dual‐target conventional real‐time RT‐PCR tests among all the tested clinical specimens with viral RNA loads of 3.48 × 10^5^−3.42 × 10^2^ copies/ml before pooling, and the PPA was 100% (Tables 2 and 3), indicating that the POC real‐time RT‐PCR tests have comparable detection sensitivity to the conventional type. When the 5‐specimen pooled strategy was applied, both the Xpert Xpress and cobas Liat platforms also exhibited a PPA of 100% among specimens with high and intermediate viral loads ranging from 3.48 × 10^5^ to 3.44 × 10^3^ copies/ml before pooling compared to the results of individual testing by the same platform. For the specimens with low viral RNA loads (1.59 × 10^3^–3.42 × 10^2^ copies/ml before pooling), the Xpert Xpress had a PPA of 100%, whereas the cobas Liat failed to detect 1 out of the 16 5‐specimen pooled samples with a PPA of 93.75% (Tables 2 and 3). The specimen that was missed by the cobas Liat had Ct values of 33.82 and 37.97 in the E and N2 assays, respectively, when tested individually.

**TABLE 2:** Performance of the Xpert Xpress real‐time RT‐PCR for detection of SARS‐CoV‐2 viruses through the individual and pooled testing strategy

Ct group of individual specimens^a^ (number of specimens)	Viral RNA quantity of the individual specimens (copies/ml)^c^	Results of POC real‐time RT‐PCR (Xpert Xpress)					
		Individual testing		Positive percent agreement^b^	5‐specimen‐pooled testing		Positive percent agreement (%)^d^
		Positive	Negative		Positive	Negative	
28–30 (*n* = 11)	3.48 × 10^5^–7.47 × 10^4^	11	0	100	11	0	100
31–34 (*n* = 23)	3.46 × 10^4^–3.44 × 10^3^	23	0	100	23	0	100
35–37 (*n* = 16)	1.59 × 10^3^–3.42 × 10^2^	16	0	100	16	0	100

^a^ The Ct group was classified by those determined by the conventional N2 real‐time RT‐PCR assay.

^b^ Positive percent agreement was determined by comparing results of individual testing to those of dual‐target conventional real‐time RT‐PCR.

^c^ The amount of viral RNA copies was determined based on the standard curve of conventional N2 real‐time RT‐PCR assay.

^d^ Positive percent agreement was determined by comparing results of the 5‐pooled specimens with the respective positive specimen detected individually.

**TABLE 3:** Performance of the cobas Liat real‐time RT‐PCR for detection of SARS‐CoV‐2 viruses through the individual and pooled testing strategy

Ct group of individual specimens^a^ (number of specimens)	Viral RNA quantity of the individual specimens (copies/ml)^c^	Results of POC real‐time RT‐PCR (Xpert Xpress)					
		Individual testing		Positive percent agreement^b^	5‐specimen‐pooled testing		Positive percent agreement (%)^d^
		Positive	Negative		Positive	Negative	
28–30 (*n* = 11)	3.48 × 10^5^–7.47 × 10^4^	11	0	100	11	0	100
31–34 (*n* = 23)	3.46 × 10^4^–3.44 × 10^3^	23	0	100	23	0	100
35–37 (*n* = 16)	1.59 × 10^3^–3.42 × 10^2^	16	0	100	15	1	93.75

^a^ The Ct group was classified by those determined by the conventional N2 real‐time RT‐PCR assay.

^b^ Positive percent agreement was determined by comparing results of individual testing to those of dual‐target conventional real‐time RT‐PCR.

^c^ The amount of viral RNA copies was determined based on the standard curve of conventional N2 real‐time RT‐PCR assay.

^d^ Positive percent agreement was determined by comparing results of the 5‐pooled specimens with the respective positive specimen detected individually.

Comparing the Ct values of the 5‐specimen pooled samples to those of the respective positive specimens that were tested individually by the same platform showed that the average Ct values obtained through the pooling strategy significantly increased by 2.8 (*p* < 0.01) and 2.5 (*p* < 0.0001) in the E and N2 assays for Xpert Xpress, respectively (Figure 2B), and 1.8 (*p* < 0.0001) for cobas Liat (Figure 2C).

## 4 DISCUSSION

Because COVID‐19 vaccines are not as effective at completely preventing SARS‐CoV‐2 from infecting and spreading among the human population^19^ and because transmission through asymptomatic or presymptomatic virus‐infected cases continuously occurs in the community,^20^ a mass testing strategy is still a useful countermeasure to block the viral transmission route and prevent the spread of viruses.^21^, ^22^ Early identification of these hidden individuals before they can further transmit SARS‐CoV‐2 to other susceptible people can have a very large positive impact on the development of surge epidemics. For this purpose, the use of POC‐based real‐time RT‐PCR tests in addition to the conventional‐type platform may provide rapid, sensitive, and specific SARS‐CoV‐2 detection,^23^ and the pooled‐specimen screening strategy is an effective way to increase testing capacity and save substantial resources, making laboratory diagnosis sustainable.^24^

In this study, we comprehensively evaluated the performance of one dual‐target conventional and two POC real‐time RT‐PCR tests for the detection of SARS‐CoV‐2 from nasopharyngeal or oropharyngeal specimens in either a separate or a 5‐specimen pooling manner. Xpert Xpress and cobas Liat are two of the rapid real‐time RT‐PCR platforms offering sample‐to‐answer detection of SARS‐CoV‐2 in 50 min and 20 min, respectively, with detection limits of 100 copies/ml (Xpert Xpress,^25^) and 12 copies/ml (cobas Liat, product datasheet). The dual‐target conventional real‐time RT‐PCR assay, which serves as the reference method, has been reported to exhibit detection limits of 3.9 and 5 copies/reaction for the E^16^ and N2^26^ assays, respectively. The use of these real‐time RT‐PCR assays with high detection sensitivity to overcome the potential effect of sample dilution makes pooled testing a possible efficient strategy for virus detection in a timely manner. Theoretically, 5‐specimen pooled testing would have 5‐fold dilution for each positive sample, leading to an increased Ct value of 2.32 compared to the value when the positive specimen is tested individually. Therefore, this testing strategy is sometimes challenging since it may possibly cause false negative results, especially for specimens whose diluted viral load is around or lower than the detection limit (eg Ct 35–40) of real‐time RT‐PCR assays.^27^ The US FDA also generally recommends that pooled testing with ≥85% positive agreement compared with the same platform when specimens are tested individually is acceptable.

Regarding the dilution effect caused by pooled testing, previous studies have reported that pooling had a change in Ct value of 2.0 in pools of 4 and of 2.9 in pools of 6 by using the Xpert Xpress platform, and samples with individual Ct values of 20–28 can be reliably detected by the two pooled testing strategies.^28^ Another study reported that samples with input viral loads ranging from approximately 2.85 to 938 copies/mL (Ct values of 23–35) could also be correctly identified by Xpert Xpress, although the tested sample number was only 5.^29^ In this study, 5‐specimen pooling showed an increase in the Ct value of 1.7–2.0 in conventional real‐time RT‐PCR, 2.5–2.8 in Xpert Xpress and 1.8 in cobas Liat. The concordance between the results of individual and 5‐specimen pooled sample testing of nasopharyngeal and oropharyngeal specimens that contained viral RNA loads in the range of 3.48 × 10^5^ to 3.42 × 10^2^ copies/ml obtained by each of the three evaluated real‐time RT‐PCR tests was 100%–93.75%. These experimental data highlighted that the Ct values increased by the pooling strategy were close to those of the theoretical calculation, and this increase could be used to estimate the performance of a given real‐time RT‐PCR platform with a known detection limit when applied in pooled testing. Furthermore, it shows that not only conventional but also POC real‐time RT‐PCR platforms can be efficiently applied in SARS‐CoV‐2 laboratory diagnosis either for individual testing or pooled testing; before, the latter was empirically considered infeasible since the inferior sensitivity of commercial POC real‐time RT‐PCR tests due to the combination of nucleic acid extraction and real‐time RT‐PCR in a closed system was usually criticized.

The 5‐specimen pooled testing strategy reported in this study showed that an acceptable balance between the quantity of simultaneously tested samples and the analytical sensitivity for SARS‐CoV‐2 can be achieved. Among specimens with high and intermediate levels of viral loads (Ct 28–34), the positive percent agreement between the three evaluated real‐time RT‐PCR platforms was 100%. For separate positive sample panels for dual‐target conventional real‐time RT‐PCR and cobas Liat, each showed 1 out of the 16 with low viral loads (Ct 35–37) as undetectable through 5‐specimen pooled testing. This missed detection may not cause virus spread since infected patients whose respiratory specimens have Ct values greater than 33–34 were not contagious.^30^ However, caution should be taken if the patient is in the early stage after virus exposure, as their viral loads may subsequently elevate. Apart from loss of sensitivity, one of the other limitations of pooled testing is that positive data confirmation may be delayed due to deconvoluting and retesting all the specimens individually when the corresponding sample pool was identified as positive. Since the two POC real‐time RT‐PCR tests evaluated in this study could both be finished within 60 min, we proposed that they are beneficial to overcome these data reporting issues. Another recommendation could be to implement pooled testing in low virus prevalence scenarios to minimize the requirement for a second round of individual testing. In summary, we demonstrate that the use of a rapid and sensitive real‐time RT‐PCR platform will maximize testing capacity and resource conservation through a pooled testing strategy with a group size of 5.

Limitations of this study include the small numbers of specimens and diagnostic platforms that were enrolled and tested which may bias the interpretation of the evaluation data. Future works by incorporating more representative clinical specimens are suggested to obtain a better scenario for application of pooled testing strategy in SARS‐CoV‐2 nucleic acid detection.

## 5 CONCLUSION

We demonstrated that the pooling strategy is practical for routine SARS‐CoV‐2 diagnosis by using such real‐time RT‐PCR tests without significant loss of detection sensitivity, even for specimens with low RNA loads. Furthermore, the applicability of POC real‐time RT‐PCR platforms in either individual or pooled testing strategies was particularly highlighted since they can produce comparable data to that of the conventional test in a shortened time period. Our data serve as proof of concept supporting the use of various real‐time RT‐PCR platforms in the routine detection of SARS‐CoV‐2 from pooled specimens and the feasibility of enforcing POC real‐time RT‐PCR tests in local laboratories for efficient and rapid COVID‐19 diagnosis.

## CONFLICT OF INTEREST

The authors declare that they have no conflicts of interest.

## Data Availability

All data generated or analyzed during this study are included in this published article.
